# Hydroquinone predisposes for retinal pigment epithelial (RPE) cell degeneration in inflammatory conditions

**DOI:** 10.1007/s12026-022-09300-0

**Published:** 2022-06-04

**Authors:** Niina Bhattarai, Maria Hytti, Mika Reinisalo, Kai Kaarniranta, Yashavanthi Mysore, Anu Kauppinen

**Affiliations:** 1 School of Pharmacy, Faculty of Health Sciences, University of Eastern Finland, 70210 Kuopio, Finland; 2 Department of Ophthalmology, Institute of Clinical Medicine, University of Eastern Finland, 70210 Kuopio, Finland; 3 Department of Ophthalmology, Kuopio University Hospital, 70210 Kuopio, Finland

**Keywords:** Antioxidants, ARPE-19, Hydroquinone, IL-1α, PEDF, ROS, VEGF

## Abstract

In addition to hypoxia, inflammation is capable of inducing vascular endothelial growth factor (VEGF) expression in human retinal pigment epithelial (RPE) cells. Excessive levels of VEGF promote choroidal neovascularization and thereby contribute to the pathogenesis of wet age-related macular degeneration (AMD). Intravitreal anti-VEGF injections ameliorate pathological vessel neoformation in wet AMD but excessive dampening of VEGF can result in a degeneration of the RPE. In the present study, we induced VEGF production by exposing human ARPE-19 cells to the pro-inflammatory IL-1α and subsequently to hydroquinone, a component of tobacco smoke that is a major environmental risk factor for AMD. Effects were monitored by measuring the levels of VEGF and anti-angiogenic pigment epithelium-derived factor (PEDF) using an enzyme-linked immunosorbent assay (ELISA) technique. In addition, we measured the production of reactive oxygen species (ROS) using the 2′,7′-dichlorofluorescin diacetate (H2DCFDA) probe and studied the effects of two anti-oxidants, ammonium pyrrolidinedithiocarbamate (APDC) and N-acetyl-cysteine (NAC), on VEGF production. Cellular and secreted VEGF as well as secreted PEDF levels were reduced at all tested hydroquinone concentrations (10, 50, or 200 µM); these effects were evident prior to any reduction of cell viability evoked by hydroquinone. Cell viability was carefully explored in our previous study and verified by microscoping in the present study. APDC further reduced the VEGF levels, whereas NAC increased them. The 50 μM concentration of hydroquinone increased ROS production in ARPE-19 cells primed with IL-1α. Hydroquinone disturbs the regulatory balance of VEGF and PEDF in inflammatory conditions. These data support the idea that hydroquinone mediates RPE degeneration by reducing VEGF levels and may predispose to dry AMD since VEGF is as well important for retinal integrity.

## Introduction

Tobacco smoke is a well-known risk factor contributing to the development of age-related macular degeneration (AMD) [1]. AMD is a multifactorial disease in which retinal pigment epithelial (RPE) cells, photoreceptors, and choriocapillaris degenerate causing an impairment of central vision [2–4]. Wet (exudative) AMD is primarily characterized by choriocapillaris degeneration and choroidal neovascularization, as well as hemorrhages, edema, and exudate formation in the retina with the subsequent degeneration of the retinal pigment epithelium [5–9]. In dry (atrophic) AMD, the RPE degeneration precedes the decline of the photoreceptors and choriocapillaris leading eventually to a larger regional RPE cell loss [4, 5, 9, 10]. RPE cells are essential for vision since they maintain the functionality of photoreceptor cells [2, 4, 11]. Due to the constant light exposure, high oxygen consumption, and recycling of photoreceptor outer segments, RPE cells are constantly subjected to high oxidative stress, and at risk of oxidative damage and inflammation, i.e., they are central players in the pathogenesis of AMD [2, 4]. RPE cells are capable of secreting pro-inflammatory cytokines, such as IL-1α, that further contribute to the retinal degeneration, e.g., by inducing a local angiogenic response as has been shown in a mouse model and in cultured RPE cells [12–14]. RPE-derived IL-1α also serves as an alarmin that promotes the release of other pro-inflammatory cytokines and the production of nucleotide-binding domain, leucine-rich repeat, pyrin domain 3 (NLRP3) receptor protein, pro-caspase-1, and pro-IL-1β that are essential for the NLRP3 inflammasome activation seen in the eyes of both dry and wet AMD patients [14–16].

VEGF is a critical mediator of choroidal neovascularization in wet AMD and known to be induced by cigarette smoke [2, 6, 7, 17, 18]. Intravitreal anti-VEGF injections are administered as treatment to patients with wet AMD [5–9], but excessive anti-VEGF therapy can increase the risk of RPE degeneration since VEGF is also important for retinal integrity and for the survival and the functionality of its cells and the choriocapillaris [19–21]. Pigment epithelium-derived factor (PEDF) is an anti-angiogenic protein that reduces neovascularization by regulating VEGF expression and by competing for receptor binding [7, 22, 23]. In addition, PEDF has been reported to have several beneficial properties; it is an antioxidant and it can enhance mitochondrial activity and improve the visual cycle, as well as supporting cell survival and proliferation [24, 25]. Collectively, both VEGF and PEDF have important protective functions in the retina and their excessive reduction may have detrimental effects [8, 19, 21, 24–26].

Hydroquinone is a toxic compound present in tobacco smoke and it is known to exert many detrimental effects on RPE cells, such as a reduction in cell size and the promotion of oxidative stress, apoptosis, and nuclear condensation [18, 27]. Since its effects on angiogenic factors are not fully understood, we examined the role of hydroquinone on the production of VEGF and PEDF levels in human RPE cells after exposing the cells to IL-1α [28]. Main data were collected using undifferentiated ARPE-19 cells that serve as an initial experimental model in general. Moreover, central results were confirmed using differentiated ARPE-19 cells. In subsequent studies, we need to confirm our findings using primary human RPE cells or induced pluripotent stem cell (iPSC)-derived RPE cells but at this point, our results suggest that by significantly reducing the levels of VEGF, hydroquinone is a potential risk for RPE cell degeneration.

## Materials and methods

### Cell culture and treatments

Experiments were performed using human ARPE-19 cells purchased from the American Type Culture Collection (ATCC). Cells were cultured in Dulbecco’s modified Eagle’s medium (DMEM) with a nutrient mixture F-12 1:1 (Life Technologies, Paisley, UK), penicillin 100 U/ml and streptomycin 100 μg/ml (Life Technologies, Grand Island, NY, USA), 2 mM L-glutamine (Life Technologies, Paisley, UK), and 10% fetal bovine serum (GE Healthcare Life Sciences, South Logan, UT, USA). Experiments were conducted in a medium including L-glutamine and antibiotics but without serum supplementation. L-Glutamine was added into the medium separately just prior to cell exposures, as described previously [28].

In the experiments, cells were seeded at a density of 200,000 cells/well onto 12-well plates (Costar, Corning Inc., Kennebunk, ME, USA) except in experiments involving ROS measurements where the density was 15,000 cells/well in 96-well plates (Costar, Corning Inc., NY, USA). All cell cultures were incubated for three days at + 37 °C, 5% CO_2_. Thereafter, cells were washed once with serum-free medium and primed using IL-1α (4 ng/ml; R&D Systems, Minneapolis, MN, USA) for 24 h at + 37 °C, 5% CO_2_. IL-1α was removed by washing cells with serum-free medium before an exposure to 10 μM, 50 μM, or 200 μM hydroquinone (HQ; Sigma-Aldrich, Saint Louis, MO, USA) for 15 min (lactate dehydrogenase [LDH] measurement), for 15 min or 1 h (ROS detection), or for 18 h (VEGF and PEDF determination) at + 37 °C, 5% CO_2_. Ammonium pyrrolidinedithiocarbamate (APDC, 2 μM; Sigma-Aldrich, St. Louis, MO, USA) was added 5 min and N-acetyl-cysteine (NAC, 5 mM; Sigma-Aldrich, St. Louis, MO, USA) 1 h before hydroquinone treatment, as described previously [28]. Hydroquinone was dissolved in the medium just before each experiment. After the treatments, cells were visually examined under an inverted light microscope (Axio Vert A1 Zeiss microscope with an AxioCam MRm camera and Zen 2011 program; Carl Zeiss Microscopy GmbH, Jena, Germany) before cell culture medium samples were collected into microtubes and centrifuged at 381 × g for 10 min, + 4 °C (Biofuge Fresco Heraeus Instruments, Newport Pagnell, UK). LDH was measured immediately after the sample collection and then the medium samples were stored at − 20 °C prior to the analyses (VEGF, PEDF). After removal of the medium, cells were kept on ice, rinsed once with ice-cold Dulbecco’s phosphate-buffered saline (DPBS; Life Technologies, Paisley, UK), scraped and collected in DPBS into microtubes, and centrifuged at 16,090 × g for 10 min, + 4 °C. Cell pellets were stored at − 80 °C until used in the ELISA assay (VEGF). ROS production was determined directly on cell culture plate.

ARPE-19 cells were differentiated by seeding them at a density of 200,000 cells/well onto 12-well plates and growing for 4 weeks in Dulbecco’s modified Eagle’s medium (DMEM) with a nutrient mixture F-12 1:1 (Life Technologies, Paisley, UK) supplemented with penicillin 100 U/ml and streptomycin 100 μg/ml (Life Technologies, Grand Island, NY, USA), 2 mM L-glutamine (Life Technologies, Paisley, UK), and 1% fetal bovine serum (GE Healthcare Life Sciences, South Logan, UT, USA). Culture medium was changed three times per week. Thereafter, differentiated ARPE-19 cells were washed once with serum-free medium and primed using IL-1α (4 ng/ml; R&D Systems, Minneapolis, MN, USA) for 24 h at + 37 °C, 5% CO_2_. IL-1α was removed by washing cells with serum-free medium and cells were exposed to 10 μM or 200 μM hydroquinone (HQ; Sigma-Aldrich, Saint Louis, MO, USA) in serum-free medium for 18 h at + 37 °C, 5% CO_2_ (for VEGF and LDH determination).

### Enzyme-linked immunosorbent assay (ELISA)

An enzyme-linked immunosorbent assay (ELISA) was used to analyze the secretion of VEGF and PEDF from medium samples as well as VEGF from cell lysates using commercial kits and following the manufacturers’ instructions. VEGF was measured using the DuoSet, Human VEGF kit (R&D Systems, Minneapolis, MN, USA) and PEDF using the Human Serpin F1/PEDF DuoSet ELISA kit (R&D Systems, Minneapolis, MN, USA). In the detection of intracellular VEGF, depending on the experiment, cells scraped from one or two wells of a 12-well plate were lysed with 40 μl of lysis buffer (1 × ; Cell Lysis Buffer 10 × , Cell Signaling Technology, Leiden, Netherlands). The cells were incubated in the lysis buffer on ice for 5 min, sonicated for 3 × 10 s, and centrifuged at 16,090 × g for 10 min at + 4 °C. Protein levels were measured using a protocol based on the Bradford method [29]. Briefly, the measurement was performed on a 96-well plate (Greiner Bio-One GmbH, Frickenhausen, Germany), and bovine serum albumin fraction V (BSA 1 mg/ml; Roche, Mannheim, Germany) ranging from 0.5 μg/μl to 3.5 μg/μl served as a standard curve. Bradford solution was added into each well (200 µl/well) and the plate was shaken. Absorbance values were detected at the wavelength of 595 nm using a spectrophotometer Bio-Rad Model 550 and Microplate Manager 5.2 program (Bio-Rad Laboratories Inc., Hercules, CA, USA). VEGF levels were determined using the entire cell lysate by the ELISA, and results were normalized to the protein levels of the respective sample.

Absorbance values of samples were measured using a spectrophotometer (Bio-Rad Model 550 and Microplate Manager 5.2 program; Bio-Rad Laboratories Inc., Hercules, CA, USA) at the wavelength of 450 nm and with the reference wavelength of 655 nm.

### Detection of reactive oxygen species (ROS)

After an exposure of cells to hydroquinone, cells were washed once with serum-free medium, and 5 μM 2′,7′-dichlorofluorescin diacetate probe (H2DCFDA; Molecular probes, Life Technologies, Eugene, OR, USA) was added for 1 h and then the cells were incubated in the dark at + 37 °C, 5% CO_2_. Thereafter, the cells were washed twice with 100 μl DPBS (Life Technologies, Paisley, UK) followed by the addition of 100 μl DPBS, and fluorescence intensity (excitation 488 nm/emission 528 nm) was measured using a BioTek Cytation3 imaging reader with Gen-5 3.03 program (BioTek, Instruments Inc., Winooski, VT, USA).

### Cellular viability

Lactate dehydrogenase (LDH) release, which is indicative of cell membrane rupturing, was measured from medium samples immediately after the sample collection. LDH was detected using a commercial assay according to the manufacturer’s instructions (CytoTox96® Non-Radioactive Cytotoxicity Assay; Promega, Madison, WI, USA). Absorbance values were detected using a spectrophotometer (BioTek, ELx808 with the Gen-5 2.04 program; Instruments Inc., Winooski, VT, USA) at the wavelength of 490 nm.

### Statistical analyses

The GraphPad Prism program 7.04 (GraphPad Software, San Diego, CA, USA) was used in the statistical analyses. Statistical analyses between groups were performed using the Kruskal–Wallis test and pairwise comparisons between independent groups were analyzed using the Mann–Whitney *U*-test. Results are presented as mean ± standard error of mean (SEM) and *P*-values below 0.05 were considered statistically significant.

## Results

### Hydroquinone reduces VEGF levels in ARPE-19 cells

**Fig. 1:**
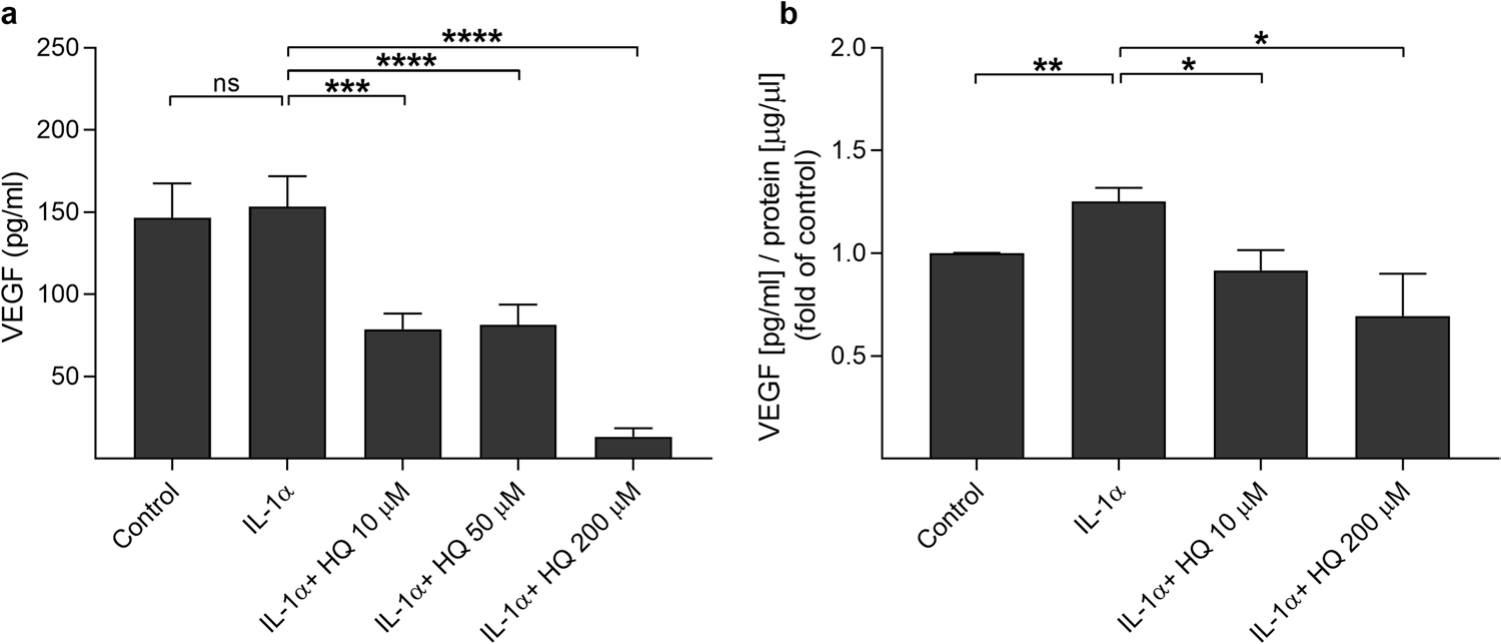
The effect of hydroquinone (HQ 10 μM, 50 μM, 200 μM; 18 h) on the secreted (**a**) and cellular (**b**) VEGF levels in IL-1α-primed ARPE-19 cells. Concentrations (pg/ml) were normalized to the protein levels of the respective sample and compared to the mean of untreated control group (**b**). Concentrations (**b**) ranged from 29.0 to 213.4 pg/ml in IL-1α, from 17.7 to 100.7 pg/ml in IL-1α + HQ 10 μM, and from 2.2 to 111.9 pg/ml in IL-1α + HQ 200 μM groups. Data were combined from 3 to 6 (**a**) or 3 to 4 (**b**) independent experiments containing 4 (**a**) or 1–2 (**b**) parallel samples per group in each experiment. Total sample numbers were as follows: 36 (**a**) or 11 (**b**) in control and IL-1α groups, 24 (**a**) or 6 (**b**) in IL-1α + HQ 10 μM or 50 μM groups, and 12 (**a**) or 6 (**b**) in the IL-1α + HQ 200 μM group. Results were analyzed using the Mann–Whitney *U*-test and shown as mean ± standard error of mean (SEM). *P < 0.05, **P < 0.01, ***P < 0.001, ****P < 0.0001. (Kruskal–Wallis test, (**a**) ****P < 0.0001, (**b**) *P < 0.05)

**Fig. 2:**
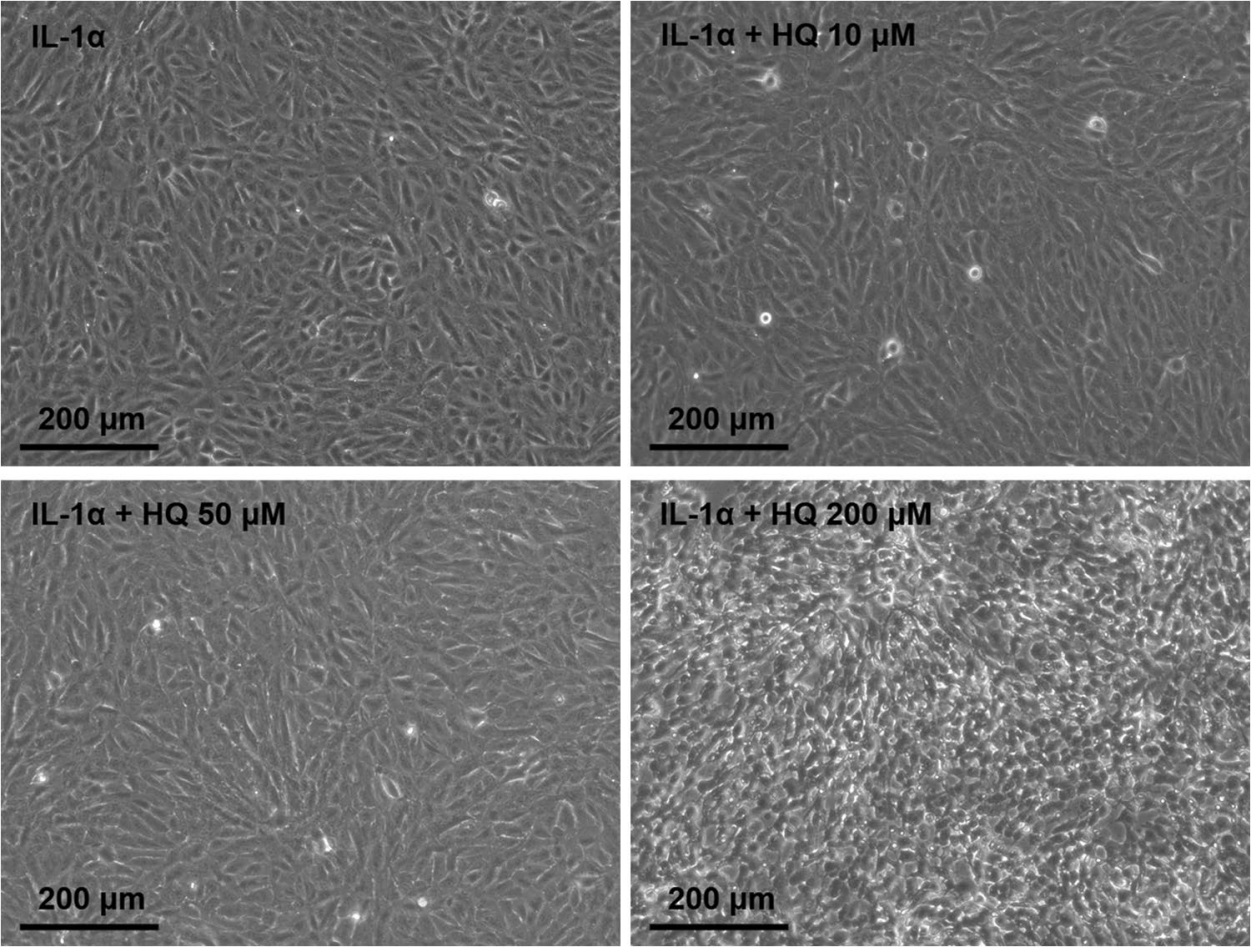
The effect of hydroquinone (HQ 10 μM, 50 μM, 200 μM; 18 h) on the viability of IL-1α-primed ARPE-19 cells visualized using an Axio Vert A1 Zeiss microscope with AxioCam MRm camera and Zen 2011 program (Carl Zeiss Microscopy GmbH, Jena, Germany)

Tobacco smoke is known to induce VEGF release in RPE cells [18], but the specific role of hydroquinone is not clear. We wanted to study its effects under the inflammatory conditions that prevail in the diseased retina. Inflammation was induced using IL-1α, which is an early inflammatory mediator contributing to the neovascularization in the retina [12, 30]. When hydroquinone was added to primed ARPE-19 cells, the levels of both secreted and cellular VEGF were significantly reduced at 10, 50, and 200 µM hydroquinone concentrations (Fig. 1). We have previously shown that only 200 µM hydroquinone is toxic to RPE cells [28]. Hydroquinone concentrations 10 μM and 50 μM were well tolerated also according to the microscopic examination, whereas cells exposed to 200 µM hydroquinone were visibly damaged (Fig. 2). Collectively, these data suggest that hydroquinone reduces VEGF levels in human RPE cells exposed to IL-1α already before cytotoxic effects emerge.

### Hydroquinone reduces the release of PEDF from IL-1α-primed ARPE-19 cells

**Fig. 3:**
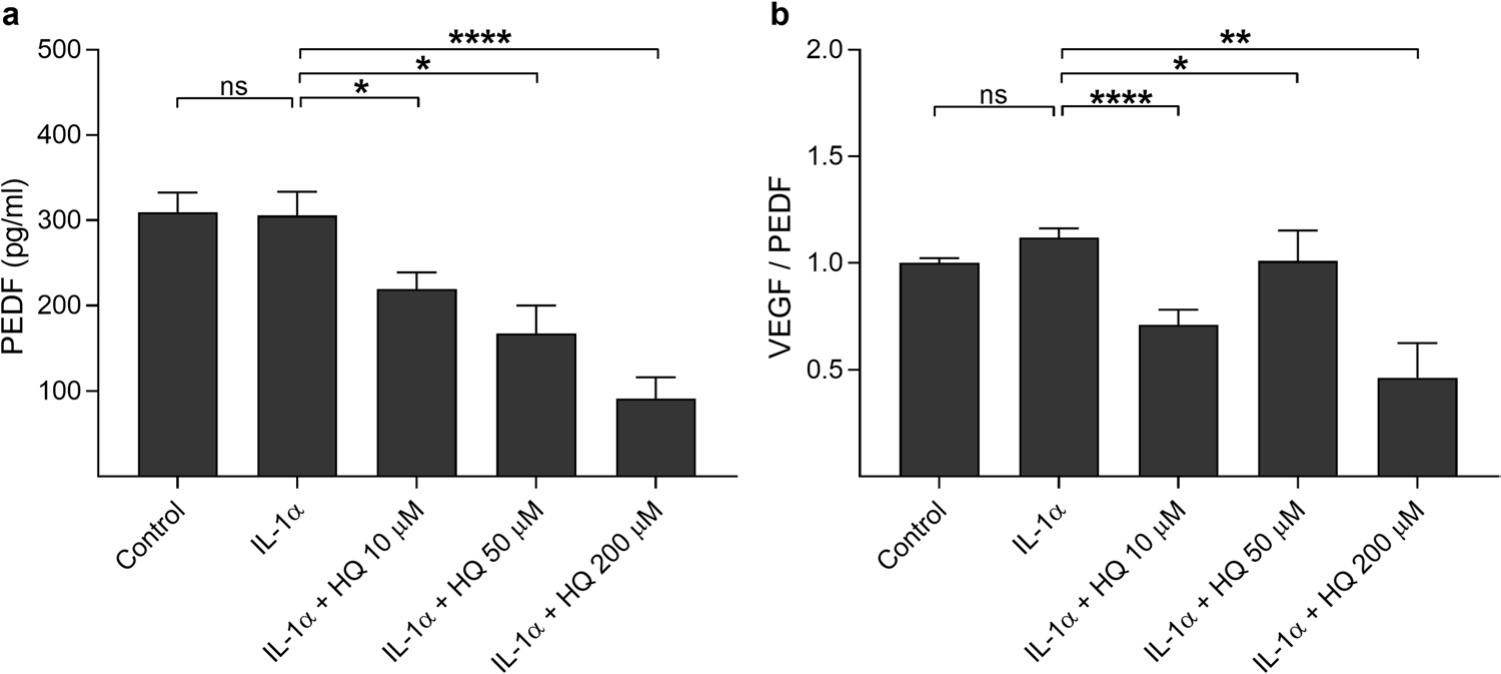
The effect of hydroquinone (HQ 10 μM, 50 μM, 200 μM; 18 h) on the PEDF secretion (**a**) and on the ratio of secreted VEGF vs. PEDF (**b**) in IL-1α-primed ARPE-19 cells. Data were combined from 3 (**a**) or 3 to 6 (**b**) independent experiments containing 3 (**a**) or 3–4 (**b**) parallel samples per group in each experiment. Total sample numbers were as follows: 18 (**a**) or 36 (**b**) in control and IL-1α groups, 9 (**a**) or 24 (**b**) in IL-1α + HQ 10 μM or 50 μM groups, and 9 (**a**) or 12 (**b**) in the IL-1α + HQ 200 μM group. Results were analyzed using the Mann–Whitney *U*-test and are shown as mean ± standard error of mean (SEM). *P < 0.05, **P < 0.01, ****P < 0.0001, ns – not significant. (Kruskal–Wallis test, (**a**) ****P < 0.0001, (**b**) ****P < 0.0001)

Since an increased ratio of the pro-angiogenic VEGF vs. the anti-angiogenic PEDF predisposes to choroidal neovascularization in the retina [23, 26], we found it important to measure also PEDF levels from culture medium samples after the exposure of the cells to hydroquinone. IL-1α treatment had no effect on the PEDF secretion or on the VEGF/PEDF ratio when compared to untreated cells (Fig. 3). In contrast, all hydroquinone concentrations significantly and concentration-dependently reduced the levels of extracellular PEDF (Fig. 3a). In addition, the ratio between VEGF and PEDF was significantly reduced (Fig. 3b). Overall, reduced PEDF levels alone would indicate a diminished anti-angiogenic effect but the concurrently reduced VEGF/PEDF ratio suggests that the cells did not need to increase PEDF production to reduce VEGF and neovascularization. On the other hand, low PEDF levels increase a susceptibility to RPE cell degeneration.

### Reduced VEGF and PEDF levels do not directly correlate with ROS production

**Fig. 4:**
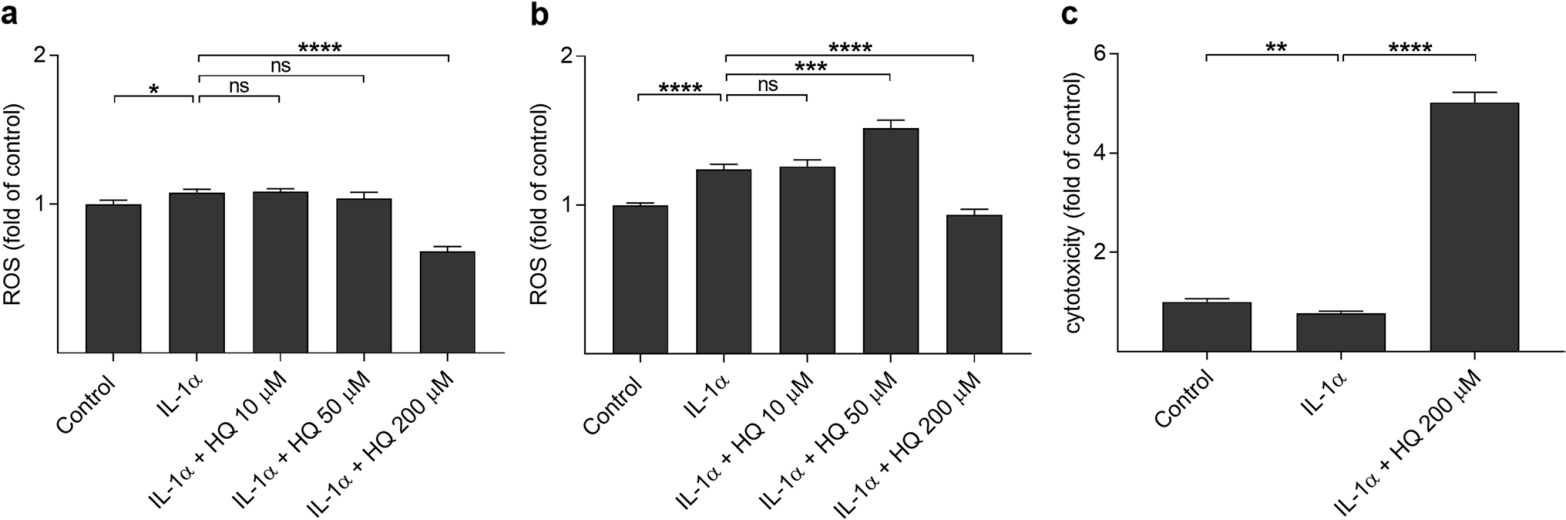
The effect of hydroquinone (HQ 10 μM, 50 μM, 200 μM) on the ROS production at 15 min (**a**) or 1 h (**b**) timepoint and on the LDH release (**c**) after 15 min exposure of IL-1α-primed ARPE-19 cells. Results were compared to the untreated control group and were combined from 3 independent experiments containing 6 (**a**–**b**) or 4 (**c**) parallel samples per group in each experiment. Total sample numbers were as follows: 18 (**a**–**b**) or 12 (**c**) in each group. Results were analyzed using the Mann–Whitney *U*-test and are shown as mean ± standard error of mean (SEM). *P < 0.05, **P < 0.01, ***P < 0.001, ****P < 0.0001, ns – not significant. (Kruskal–Wallis test, (**a**) ****P < 0.0001, (**b**) ****P < 0.0001, (**c**) ****P < 0.0001)

Since hydroquinone is a well-known pro-oxidant, and ROS have been shown to regulate VEGF production [31, 32], we determined whether hydroquinone would exert any effect on the ROS production in RPE cells upon inflammatory conditions and whether these changes would correlate with VEGF responses. IL-1α alone increased ROS production in ARPE-19 cells when compared to untreated control cells (Fig. 4a–b). Hydroquinone at the 10 μM concentration had no additional effect on that level but 50 μM hydroquinone significantly increased ROS production after a 1-h exposure in IL-1α-primed cells (Fig. 4b). ROS production was significantly reduced at the 200 µM hydroquinone concentration (Fig. 4a–b). This correlated with the evidence of cell damage in the significantly increased LDH levels (Fig. 4c). In summary, our data indicate that VEGF production is reduced by hydroquinone irrespective of the ROS production.

### NAC increases and APDC reduces VEGF release from RPE cells upon high hydroquinone exposure

**Fig. 5:**
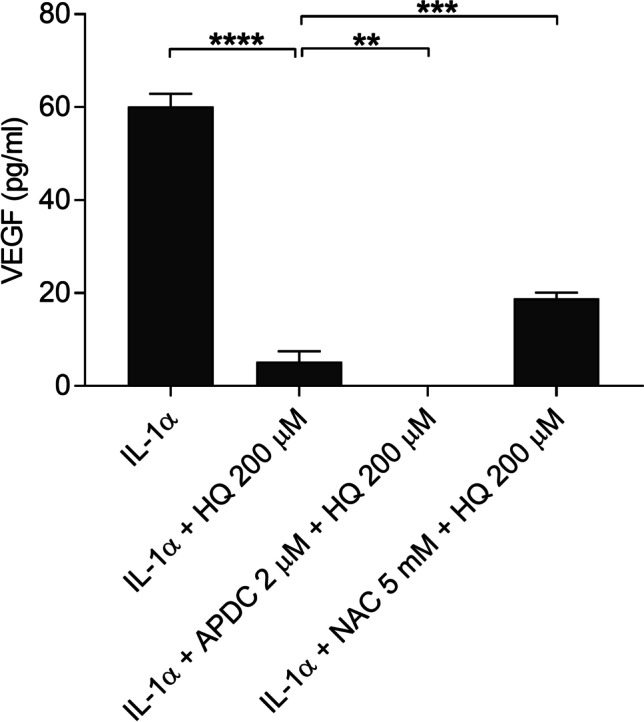
The effect of antioxidants (2 μM APDC or 5 mM NAC) on the VEGF release upon hydroquinone (HQ 200 μM; 18 h) exposure in IL-1α-primed ARPE-19 cells. Results were combined from 3 independent experiments containing 3 parallel samples per group in each experiment. Total sample number was 9 in each group*.* Results were analyzed using the Mann–Whitney *U*-test and are shown as mean ± standard error of mean (SEM). **P < 0.01, ***P < 0.001, ****P < 0.0001. (Kruskal–Wallis test, ****P < 0.0001)

We have previously shown that both NAC and APDC reduce hydroquinone-induced ROS production [27]. Since ROS were not related to VEGF response, we next tested the effect of antioxidants on cells severely damaged by hydroquinone and monitored the release of VEGF. This same model has also been used in our previous study [28]. Despite the cell damage caused by hydroquinone, we observed that the two antioxidants evoked different effects. The NADPH oxidase inhibitor APDC terminated the VEGF release altogether, whereas the glutathione precursor NAC increased it by 3.5-fold when compared to hydroquinone-treated cells without antioxidants (Fig. 5). Both changes were statistically significant and did not result from increased cytotoxicity as previously shown [28]. These data suggest that both NADPH oxidase and glutathione participate in the regulation of the VEGF response but have very different roles.

### Hydroquinone reduces VEGF release also from IL-1α-primed differentiated ARPE-19 cells

**Fig. 6:**
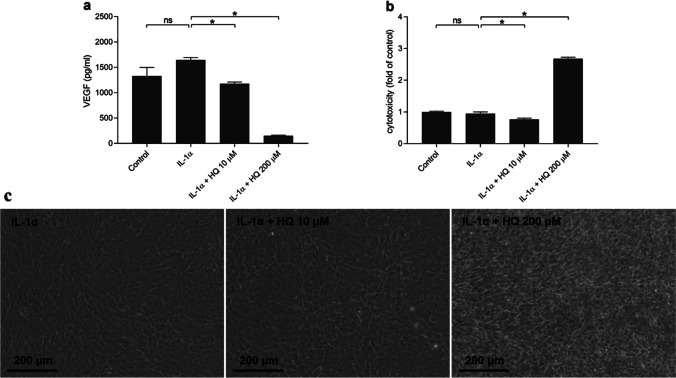
The effect of hydroquinone (HQ 10 μM, 200 μM; 18 h) on the levels of VEGF (**a**) and LDH (**b**) released by IL-1α-primed differentiated ARPE-19 cells. The condition of cells was also observed using an Axio Vert A1 Zeiss microscope with AxioCam MRm camera and Zen 2011 program (**c**). The data was collected from one experiment containing four parallel samples per group. Results were analyzed using the Mann–Whitney *U*-test and are shown as mean ± standard error of mean (SEM). *P < 0.05, ns, not significant. (Kruskal–Wallis test, (**a**) **P < 0.01, (**b**) ****P < 0.0001)

Since we have in the present study mostly used undifferentiated ARPE-19 cells, we wanted to confirm our VEGF results using differentiated ARPE-19 cells. Both 10 μM and 200 μM hydroquinone concentrations reduced VEGF release from differentiated ARPE-19 cells upon inflammatory conditions (Fig. 6a). Low level hydroquinone (10 μM) reduced but 200 μM hydroquinone significantly increased the LDH release from cells (Fig. 6b). The cytotoxicity of hydroquinone was confirmed by photographing cells under the microscope (Fig. 6c). Collectively, the response of differentiated ARPE-19 cells to hydroquinone resembles that of undifferentiated cells.

## Discussion

VEGF is detrimental in wet AMD but its excessive reduction can cause the degeneration of RPE cells and disruption of the connections between photoreceptors and choriocapillaris [8, 19, 33]. PEDF reduces angiogenesis by decreasing VEGF production and receptor binding but it is also able to confer protection on cells, e.g., from mitochondrial dysfunction and ROS production [22, 34–37]. Furthermore, if PEDF production is excessively low, this triggers pathological changes in the retina [22, 34–37]. Thus, a delicate balance between VEGF and PEDF maintains the homeostasis of angiogenesis in the eye and helps to avoid the pathogenesis of both wet and dry AMD [19–22, 38, 39]. In human RPE cells, both tobacco smoke extract and nicotine have been shown to increase VEGF expression, while nicotine also reduces PEDF levels and increases the ratio of VEGF to PEDF [18, 40]. Exogenous VEGF has been shown to protect RPE cells from tobacco smoke-induced cell death [41]. We have previously shown that IL-1α exposure for 72 h induces VEGF release from ARPE-19 cells [14]. In the present study, shorter IL-1α treatment was used to simulate pro-inflammatory conditions in cell cultures. In addition to IL-6 and IL-8 releases, IL-1α induces the production of NLRP3, pro-caspase-1 and pro-IL-1β that are essential for the NLRP3 inflammasome activation detected in the eyes of both dry and wet AMD patients [14–16, 42]. Hydroquinone reduced intra- and extracellular VEGF levels in IL-1α-treated human RPE cells, and this effect was significant already at low (10 and 50 µM) hydroquinone concentrations that did not compromise cellular viability [28]. In other publications, hydroquinone had no effect on the VEGF release when ARPE-19 cells were exposed to 10 µM hydroquinone repeatedly for five days or when cultured with 20 µM hydroquinone for 12 h [39, 43]. Still, hydroquinone reduced VEGF gene expression in ARPE-19 cells when treated with 10 μM concentration for five days [39]. Conversely, 75 μM hydroquinone was shown to increase VEGF secretion but in that study, primary human RPE cells were used [44]. It seems that the tolerance of ARPE-19 and primary human RPE cells varies depending on the conditions. For example, 125 µM hydroquinone was needed to result in significantly increased LDH release from ARPE-19 cells at 24 h, whereas significant rupturing of cell membranes was observed at 75 µM hydroquinone already in 12 h in primary human RPE cells [27, 44]. Primary cells also tend to secrete higher levels of IL-1β than ARPE-19 cells under similar conditions [16].

PEDF is a serine protease inhibitor (Serpin F1) that protects the retina from oxidative stress not only by regulating cell survival pathways but also via the expression of inflammatory and pro-angiogenic factors [45]. It has not been extensively investigated in association with hydroquinone on RPE cells but in one study in ARPE-19 cells, no change was observed on VEGF production with hydroquinone exposure while the levels of PEDF were reduced [39]. In our cell cultures, hydroquinone reduced the PEDF levels as well as the ratio of VEGF to PEDF. In the study of Pons et al., the VEGF/PEDF ratio was increased in ARPE-19 cells in cultures and the RPE/choroid tissues of mice exposed to hydroquinone. Also there, the change in the ratio mainly resulted from reduced PEDF levels [39]. In the RPE lysates of smoking AMD patients, the VEGF/PEDF ratio was increased but due to both increased VEGF and reduced PEDF [39]. Difference may result from local conditions since our cells were first exposed to the pro-inflammatory cytokine IL-1α. We have also observed that hydroquinone is very sensitive to any kind of changes in conditions [27, 28]. Despite its numerous protective effects, PEDF has been shown to enhance cartilage degeneration under inflammatory conditions [46]. Although IL-1β was used in the induction of inflammation in the osteoarthritis study [46], that cytokine can bind to the same receptor as IL-1α and thereby can be thought to evoke similar inflammatory conditions [47]. In PEDF-deficient mice, the cartilage loss was mediated by matrix metalloproteinases (MMPs) [46]. MMPs and their counter-regulators, tissue inhibitors of MMPs (TIMPs), regulate the extracellular matrix (ECM) the dysregulation of which have also been associated with the pathogenesis of AMD; for example, MMPs have been recognized as potential therapeutic targets [48]. In studies on ARPE-19 cells, repeated exposure to a non-lethal hydroquinone concentration (100 µM) or a single-dose of 100 µM hydroquinone for 6 or 18 h reduced the activity of MMP-2 and increased the accumulation of ECM proteins, effects which were associated with the progression of dry AMD [49, 50]. The effect could be prevented by overexpressing MMP-14 and TIMP-2 that regulate the activity of MMP-2 [50]. Together, these data suggest that an exposure of RPE cells to hydroquinone under inflammatory conditions may have deleterious effects on the ECM, such as a propensity for thickening of the Bruch’s membrane and the formation of sub-RPE deposits both of which are typically encountered in the pathology of dry AMD.

It was interesting to observe that hydroquinone-induced VEGF release was completely prevented by the NADPH oxidase inhibitor APDC, whereas the glutathione precursor NAC resulted in a partial restoration of VEGF production. This is in line with findings showing that hydroquinone exposure causes glutathione depletion in hepatocytes, the same effect evoked by anti-VEGF drugs in porcine RPE cells and conversely, VEGF confers protection from oxidative stress by inducing glutathione expression [51–54]. Moreover, PEDF has been shown to prevent ROS production via the activation of glutathione peroxidase in retinal pericytes when these cells were exposed to high glucose concentrations [35]. In our previous study, we demonstrated that although both APDC and NAC were capable of reducing ROS production [27], only NAC significantly and visibly increased cell viability and alleviated the DNA damage caused by 200 µM hydroquinone [28]. In turn, APDC significantly reduced the hydroquinone-induced IL-18 release in IL-1α-primed RPE cells [28], and according to our present data, it exerts a similar effect on VEGF. NAC also showed a reducing trend on the IL-18 production although the change was not statistically significant [28]. In our cell model stimulating inflammasome activation and IL-1β production in RPE cells with dysfunctional intracellular clearance, APDC reduced the release of mature IL-1β, unlike NAC which had no effect [55]. Together, the data suggest that RPE cells, which have been severely damaged either by MG-132 and bafilomycin A or hydroquinone, induce NADPH oxidase-mediated ROS production the consequences of which can be regulated by APDC [27, 28, 55]. On that basis, inhibition of NADPH oxidase-mediated ROS production would be an efficient way of reducing VEGF release and preventing neovascularisation. This concept is supported by a study showing that the downregulation of the integral subunit p22phox of the NADPH oxidase complex was able to inhibit choroidal neovascularisation in mice [56]. It also appears likely that VEGF production is not possible in highly damaged ARPE-19 cells but the production recovers when the cell viability increases, e.g., after NAC treatment [28]. The proposal is supported by the fact that along with its pro-angiogenic functions, VEGF is also an important survival factor and a critical component promoting cell survival and the maintenance of RPE integrity [19].

## Conclusion

Excessive VEGF is detrimental to the retina but an appropriate amount of VEGF as well as PEDF is beneficial for cell survival in times of oxidative stress, and thus the decline of both reduces RPE cell viability [6, 24, 25, 31, 54, 57]. Our present data suggest that hydroquinone, a component of tobacco smoke, reduces VEGF and PEDF levels and shifts the balance between VEGF and PEDF, which predispose cells to atrophy-like conditions in an inflammatory milieu. This strengthens the pathological role of tobacco, especially its hydroquinone component, in AMD and emphasizes the importance of assessing the harsh local conditions to which the cells in the retina are exposed.

## Data Availability

The data are available upon request.
